# Problems on the labour market for young Dutch cardiologists

**DOI:** 10.1007/s12471-021-01626-y

**Published:** 2021-08-23

**Authors:** M. Michels, G. Veen, J. G. Meeder

**Affiliations:** 1 Department of Cardiology, Erasmus MC, Rotterdam, The Netherlands; 2 Department of Cardiology, Amsterdam UMC, location VUmc, Amsterdam, The Netherlands; 3 Department of Cardiology, VieCuri Medical Centre, Venlo, The Netherlands

In their paper, Vorselaars et al. [1] describe the early career perspectives of young cardiologists in the Netherlands after questioning 174 young cardiologists between 2015 and 2018.

At first glance, the numbers look reassuring, as only one cardiologist is reported to be unemployed, and neither age, gender nor type of teaching hospital was found to have a significant influence on the time required to obtain a permanent position. However, the fact that after 3 years a third of the young cardiologists still have a temporary contract and that 44% describe the current job market as problematic is worrying. Furthermore, the percentage of temporary contracts among starting cardiologists has gradually increased over the last few years. More recently, unpublished data from a new questionnaire completed by young cardiologists confirm this trend and depict a worsening scenario, as the unemployment rate has increased and the number of young cardiologists with a permanent position has decreased.

In general, the problems on the labour market for medical specialists have increased over recent years. This is a very complex problem, and there is no quick and easy solution. The situation on the labour market for young cardiologists is the result of the complex interplay between the inflow of young cardiologists and outflow of retiring cardiologists and the Outline Agreement (*Hoofdlijnenakkoord*), a governmental financial agreement preventing any growth in specialist medical care.

Since 1999 the government has regulated the inflow into medical specialist training. The Advisory Committee on Medical Specialist Manpower Planning (*Capaciteitsorgaan*) advises the Minister of Health about the range of the number of training positions for all medical specialties. Consecutive Ministers of Health have chosen to allow the highest number of training positions, resulting in a high number of young specialists entering the labour market each year. The goal was to increase competition in the market and to reduce costs. This led to a peak of young medical specialists in 2017, only 5 years after the first Outline Agreement came into effect. This diametrically opposed policy of training the highest possible number of cardiologists, while having no budget to subsequently provide all of them with a workplace, is in our opinion the biggest problem. This is particularly bitter because an increasing demand for cardiologists is most likely to be expected until 2040 due to the increasing demand for cardiovascular care.

The Netherlands Society of Cardiology (NVVC) considers it its duty to improve the situation for young colleagues. The NVVC recently created a labour market task force group to obtain complete data on the number of cardiologists and job vacancies and to work together on solutions. This task force consists of both cardiologists in training and cardiologists at different stages of their carriers. Besides trying to reduce the inflow of cardiologists into training, reorganisation of the workload for cardiologists with a permanent position might create more space for younger colleagues and improve the work-life balance. As mentioned by Vorselaars et al. [1], we expect the results of the Working Group on Fellowships at the end of this year. We aim to align the number of fellowships with job vacancies for specific cardiology subspecialties. As this is especially urgent for interventional cardiology, we are working together with the training centres to reduce the number of training positions in order to improve the perspectives on the labour market for young interventional cardiologists.

On a national level the NVVC is active partner of the Dutch Association of Medical Specialists (FMS). This platform is now being used to take the problem of the increasing unemployment rate among young medical specialists to the highest level (Ministry of Health) in order to get away from the Outline Agreement. At the same time we will seek new advice from the Advisory Committee on Medical Specialist Manpower Planning to obtain a more realistic (reduced) inflow of cardiology residents in the coming years.
